# Application of a zona pellucida binding assay (ZBA) in the domestic cat benefits from the use of *in vitro *matured oocytes

**DOI:** 10.1186/1751-0147-49-28

**Published:** 2007-10-01

**Authors:** Ulrika Hermansson, Eva Axnér, Bodil Ström Holst

**Affiliations:** 1 Department of Clinical Sciences, Division of Reproduction, Swedish University of Agricultural Sciences (SLU), Box 7054, SE-750 07 Uppsala, Sweden; 2 Department of Clinical Sciences, Division of Diagnostic Imaging and Clinical Pathology, Swedish University of Agricultural Sciences (SLU), Box 7054, SE-750 07 Uppsala, Sweden

## Abstract

**Background:**

Zona pellucida binding assays (ZBAs) have proven useful in determining the fertilising ability of spermatozoa in several species. Most ZBAs use fresh or salt-stored oocytes collected from fresh ovaries but because ovaries are not easy to obtain on a regular basis, chilled and frozen-thawed ovaries have been tested, with varying results. The present study tested the hypothesis that cat spermatozoa, either fresh or frozen-thawed, can bind to homologous zona pellucida of oocytes retrieved from frozen-thawed queen ovaries to a similar extent as they can bind to the zona pellucida of fresh, *in vitro *matured oocytes.

**Methods:**

Ovaries were collected from queens after routine ovario-hysterectomy and either stored in NaCl at -20°C until use (treatment animals), or used fresh (controls). Cumulus-oocyte complexes (COCs) were retrieved by ovarian slicing from either source and used directly (immature oocytes from frozen-thawed ovaries; treatment animals) or after *in vitro *maturation (IVM) (fresh ovaries; controls) for 24 hours in TCM 199, supplemented with 1 IU hCG/mL and 0.5 IU eCG/mL and 0.5% bovine serum albumin (BSA). The oocytes were incubated for 4 hours in 5% CO_2 _in air at 38°C and 100% humidity in the presence of 5 × 10^6 ^fresh or frozen-thawed spermatozoa/mL. Representative samples of oocytes were processed for scanning electron microscopy (SEM).

**Results:**

Both fresh and frozen-thawed spermatozoa bound to the *in vitro *matured zona pellucida but significantly fewer, or no, spermatozoa bound to frozen-thawed, immature zona pellucida (P < 0.001). Also, more fresh spermatozoa than frozen-thawed spermatozoa bound to the zona pellucida (P < 0.001). The zona pellucida surface differed in morphology (SEM), with *in vitro *matured oocytes showing a dense surface with few fenestrations in contrast to their frozen-thawed, immature counterparts, where fenestrations were conspicuously larger.

**Conclusion:**

In conclusion, under the conditions of the present study, immature oocytes recovered from ovaries frozen immersed in NaCl at -20°C are less suitable for use in feline ZBA.

## Background

A combination of various *in vitro *tests is better at predicting fertility than is a single test as they can be combined to measure different attributes needed for fertilisation and early embryonic development [1]. Some laboratory tests, such as the zona pellucida binding assay (ZBA), together with tests used to investigate the penetration of the zona pellucida (ZP) and *in vitro *fertilisation (IVF), are able to test for several parameters and interpret the interaction between spermatozoa and the oocyte (for a review, see [2]). The approach of using such functional tests gives a better estimation of sperm fertilising capacity. Zona pellucida binding assays have been used to predict fertility in humans [3] and animals, for example cattle [4], dogs [5-8] and cats [9-11]. The ZBA has also been used to study how different sperm abnormalities influence fertility (for review, see [12,13]). Abnormal cat spermatozoa are capable of binding to and entering the outer ZP but are usually compromised in their ability to penetrate the inner zona and reach the perivitelline space to fertilise the oocyte [14,15].

The ability of cat spermatozoa to capacitate and acrosome react is essential for a successful zona penetration but not for zona binding [9,14] and corresponding results have been found in the female dog [8,16,17]. When the first spermatozoon makes contact with the oolemma after penetrating the ZP, the cortical reaction is elicited by the release and action of the contents of the cortical granules, which cause changes in the structure of the innermost layer of the ZP [18]. Such a zona reaction prevents the full penetration of spermatozoa through the entire ZP and thus diminishes or prevents the possibility of lethal polyspermia of the oocyte. Additional spermatozoa are thereafter named "accessory spermatozoa". Little is known about inhibition of polyspermia in cats, but any existing block is compromised during manipulation of the oocytes, including *in vitro *maturation (IVM) and IVF [18,19] and salt storage [20,21].

Cat oocytes intended for zona binding and zona penetration testing are either immature when used [22] or have been matured *in vitro *[9,10], or are used after controlled short-term chilling [23] or freezing and thawing [24-26]. Some are dead following salt storage [9,11,15,22] or freezing and thawing [27]. The use of fresh oocytes for ZBA makes the method very dependent on a predictable oocyte availability, and if it would be possible to perform successful sperm-zona binding with oocytes removed from frozen-thawed (FT) ovaries, this would considerably facilitate the ZBA, even though some of the properties of the ZP (the zona reaction ability, for instance; see above) are lost under some of these conditions. A ZBA with FT oocytes has been successfully used in female dogs [6-8] but has only been peripherally tested in cats [27]. In the study in cats, FT, immature oocytes were used in a ZBA with FT epididymal spermatozoa, and 16 out of 20 of the sperm samples bound to the ZP. However, the authors regarded the results as positive if at least one sperm bound to the ZP. The number of spermatozoa bound to each ZP was not reported. This scarcity of information calls for further studies.

The present study tested the hypothesis that cat spermatozoa, either fresh or FT, can bind to homologous ZP of oocytes retrieved from FT queen ovaries to a similar extent as they can bind to the ZP of fresh, *in vitro *matured oocytes.

## Methods

### Collection and preparation of oocytes

#### Frozen, immature oocytes

Ovaries from queens undergoing routine ovario-hysterectomy were obtained from three animal hospitals and clinics. The age of the queens was not known, and their cyclus stage varied. After surgery, the ovaries were placed in a plastic jar filled with NaCl and frozen and stored on site at -20°C, for later transport to the laboratory. Frozen ovaries were thawed at room temperature (~20°C) for 2 hours prior to use. Oocytes were obtained by mincing the ovaries under a stereo microscope with a scalpel in a Petri dish filled with 0.5% bovine serum albumin (BSA) in Tyrode's albumin lactate pyruvate (TALP) solution for washing. To remove the surrounding cumulus cells the oocytes were vortexed (VortexGenie 2, Labora, Sollentuna, Sweden) for 5 minutes. A pool of oocytes from different cats was used for each ZBA.

#### *In vitro *matured oocytes

Fresh ovaries were also recovered from ovaries from queens undergoing routine ovario-hysterectomy. Ovaries were placed in a plastic jar filled with NaCl and retrieved within 2 hours. As for the frozen, immature oocytes, a pool from different cats was used. Fresh oocytes were recovered as described above for "frozen, immature oocytes" and matured for 24 hours in TCM 199 (Earle's salts with glutamine) (Biochrom, Berlin, Germany) supplemented with 1 IU hCG/mL (Pregnyl^®^; N.V. Organon Oss, Oss, The Netherlands), 0.5 IU eCG (Folligon^®^; Intervet India Pvt Ltd, Pune, India) and 0.5% BSA [28]. Four drops of 100 μL maturation medium were placed in a Petri dish. Five oocytes per drop were added with a 0.7 mm pipette and covered with 3 mL mineral oil. The oocytes were then incubated for 24 hours in 5% CO_2 _in air at 38°C and 100% humidity, and intact oocytes were used for the ZBA.

### Collection and preparation of spermatozoa

Freshly ejaculated spermatozoa were collected by electroejaculation performed in seven cats aged between 9 and 19 months and with a weight of 3.1–5.3 kg. The semen was not pooled, but used from one cat at a time. The cats were clinically examined and anaesthetised with medetomidine (Domitor vet^®^; Orion Pharma, Orion Corp., Espoo, Finland), 0.08 mg/kg intramuscularly (i.m.); butorphanol (Torbugesic^®^; Fort Dodge Animal Health, Fort Dodge, IA, USA), 0.04 mg/kg i.m., and ketamine (Ketalar^®^; Pfizer, Inc., New York, NY, USA), 5 mg/kg. In addition, they were given meloxicam (Metacam^®^; Boehringer Ingelheim Vetmedica, Copenhagen), 0.3 mg/kg subcutaneously (s.c.), and their eyes were protected with Metocoel (CIBA Vision Corp., Duluth, GA, USA). Atipamezol (Antisedan vet^®^; Orion Pharma, Orion Corp., Espoo, Finland), 0.2 mg/kg s.c., was used to reverse the anaesthesia. Semen was collected in a pre-warmed Eppendorf tube by electroejaculation with a 50 Hz sine-wave electroejaculator (P-T Electronics, Boring, OR, USA). A total of 80 electric stimuli were given, at 2–5 volts for each ejaculate [29]. The collected semen was resuspended in 200 μL Tris (3.025% Tris, 1.7% citric acid, 1.25% fructose, 0.06% Na-benzylpenicillin and 0.1% streptomycin sulphate) and the motility assessed. Electroejaculation was performed with permission from the local ethics committee and the Swedish Animal Welfare Agency. The cat owners had given their informed consent.

Epididymal spermatozoa collected from the caudae epididymides obtained from eight tomcats neutered at a local animal hospital and from one cat neutered at our fertility clinic were frozen. The age and reproductive status of these cats were unknown. The caudae epididymides were dissected free from the testes. Epididymal spermatozoa were released by mincing the caudae in 200 μL Tris at 38°C in an Eppendorf tube. After 10 minutes' incubation at 38°C the epididymal tissue was removed with forceps and the motility of the suspended spermatozoa assessed. The spermatozoa were frozen according to a previously described protocol [30]. In brief, spermatozoa were extended in two steps prior to freezing using Uppsala-Equex-2 (UE-2) extenders [31]. For each freezing procedure, a pool of semen from at least two males was used. After we determined sperm concentration, the sample was extended with Uppsala-Equex-1 (UE-1) to ~50 × 10^6 ^spermatozoa/mL, and placed in a bench cooler to reach 4°C in about 45 minutes. After 60 minutes in the cooler, UE-2 was added to the sperm suspension to reach a final sperm concentration of ~25 × 10^6 ^spermatozoa/mL. The spermatozoa were loaded in 0.25 mL plastic straws that were cut to contain ~0.06 mL. Before loading the extended spermatozoa, 10 μl of a 1:1 mixture of extenders 1 and 2 was drawn into the straws to completely fill the cotton plug at the top of the straw and prevent spermatozoa from becoming lost in the plug during filling. At freezing, the straws were lowered into an Apollo SX-18 LN_2 _tank with a level of 15–18 cm of LN_2 _(MVE Cryogenetics^®^, New Prague, MN, USA) in three steps, with the top of the goblets held first at 7 cm below the opening of the tank for 2 minutes and then at 13 cm for 2 minutes and finally at 20 cm below the opening for 1 minute, before plunging them into the liquid nitrogen. The straws were thawed in a water bath at 37°C for 15 seconds and emptied into a tube containing 65 μL of the thawing extender at 37°C. The spermatozoa were then kept dark at 38°C for 5 minutes and thereafter assessed for motility and concentration.

The percentage of motile spermatozoa was 73.8 ± 14.1% (mean ± standard deviation, SD) and 42.5 ± 11.6%, for fresh and FT samples, respectively (P < 0.05). The spermatozoa were cleansed for the ZBAs either by centrifugation (800 × g for 10 minutes) through a Percoll gradient (35–70%; Amersham Biosciences AB, Uppsala, Sweden) (electroejaculated spermatozoa and FT epididymal spermatozoa, used with fresh, *in vitro *matured oocytes; experiment A) or by simple centrifugation and washing (electroejaculated spermatozoa and FT epididymal spermatozoa, 700 × g, for 6 minutes; experiment B, used for zona binding with FT oocytes) at room temperature. The spermatozoa were always resuspended with a modified Tyrode's solution (Fert-TALP) [32] to a final concentration of 5 × 10^6 ^spermatozoa/mL, assessed with a Bürker chamber. Aliquots of the sperm preparations were placed on a Makler chamber at 38°C to subjectively assess their progressive motility under a phase-contrast microscope at 200 × magnification.

### Experimental design

The study included two experiments (A and B), in which fresh, electroejaculated (experiment A) or FT, epididymal cat spermatozoa (experiment B) were ZBA-tested using either *in vitro *matured (controls) or FT (treatment animals) ZP. The ZBAs were replicated four times with either oocyte type for each experiment.

### Sperm-zona pellucida binding assay

Frozen-thawed oocytes were used directly, while fresh oocytes were *in vitro *matured before they were used for the ZBA. Two to eight ovaries were used for each ZBA. Four drops of 50 μL sperm suspension with a concentration of 5 × 10^6 ^spermatozoa/mL were placed in a Petri dish. Five oocytes per drop were added with a 0.7 mm pipette and covered with 3 mL of mineral oil. The number of Petri dishes prepared for each ZBA varied depending on the amount of oocytes that were available. Sperm-oocyte complexes were then incubated for 4 hours in 5% CO_2 _in air at 38°C and 100% humidity. After incubation the sperm-oocyte complexes were pipetted three times with a 0.7 mm pipette in 100 μL droplets of phosphate-buffered saline (PBS) with 0.5% BSA to remove loosely attached spermatozoa. Before evaluation the complexes were stained for 15 minutes in a solution of 30 μL propidium iodide in 500 μL PBS with 0.5% BSA, at 38°C. The sperm-oocyte complexes were placed on a glass slide and slightly compressed by a coverslip, with a dot of a mixture of paraffin wax and vaseline in each corner. The spermatozoa bound to the ZP were counted with epifluorescence ultraviolet (UV) illumination on a Leitz Dialux 20 microscope (× 400) (Wetzlar, Germany).

### Scanning electron microscopy of the zona pellucida

Representative oocytes (n = 27; *in vitro *matured or FT, before and after ZBA) were immersion-fixed in a 2% solution of glutaraldehyde in 0.1 M sodium cacodylate buffer. Following a secondary fixation with 2% osmium tetroxide, the oocytes were dehydrated in increasing concentrations of acetone. After being subjected to mount drying, the oocytes were mounted on stubs using carbon glue, and sputtered with platinum/palladium. Examination of the outer ZP was done using a SEM JEOL 6320F scanning electron microscope (JEOL Ltd, Akishima, Tokyo, Japan). Digital images were collected at 5 KV and computer-stored using Semafore software (JEOL Ltd, Akishima, Tokyo, Japan).

### Statistical analyses

Statistical analyses were performed using analysis of variance (ANOVA, the MIXED procedure) in the SAS program (SAS Institute Inc., Cary, NC, USA). The analyses were based on data in which each oocyte constituted one observation. Logarithmic transformation was used to obtain a more normal distribution. The statistical models included the fixed effects of ZP status (*in vitro *matured or FT) and spermatozoa status (fresh or FT) and the interactions between ZP and spermatozoa status. The random effect of replicate, nested within the combination between ZP and spermatozoa, was also included in the statistical model. Least square means (LSMs) were estimated and pair-wise tests of significance were performed for the differences between the estimated LSMs. The LSM for number of attached spermatozoa obtained from the ANOVA result was antilogged before presentation. Differences in the proportion of oocytes with attached spermatozoa (within ZP status and within spermatozoa status) were analysed with Fisher's exact test (PROC FREQ). P-values < 0.05 were considered statistically significant. Values are presented as means ± SD.

## Results

**Figure 1:**
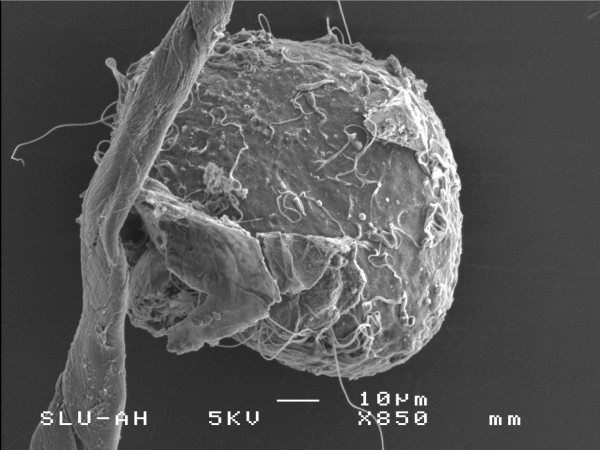
Scanning electron micrograph of fresh spermatozoa bound to a frozen-thawed ZP.

Both fresh and FT spermatozoa bound to the ZP of *in vitro *matured oocytes (Table 1, Figure 1). The percentage of binding of electroejaculated spermatozoa was higher in *in vitro *matured ZP than in the immature, FT ZP (P < 0.05). Frozen-thawed epididymal spermatozoa bound to *in vitro *matured ZP but not to FT ZP (P < 0.001). Also, comparatively more fresh, electroejaculated than FT spermatozoa bound to the ZP (P < 0.001), with barely one fresh spermatozoon bound per FT ZP. Overall, binding was significantly different between *in vitro *matured and immature, FT ZP (P < 0.05).

**Table 1:** Zona pellucida (ZP) binding shown as percentages of oocytes with bound spermatozoa and number of spermatozoa bound per oocyte (means ± standard deviation (SD)) using fresh, electroejaculated (experiment A) and frozen-thawed (FT) epididymal (experiment B) cat spermatozoa incubated with *in vitro *matured or FT homologous oocytes. For the number of spermatozoa bound to oocytes, the mean value shown is the antilogged LSM.

Exp.	Spermatozoa	*In vitro *matured oocytes			Immature, FT oocytes		
		n	ZP binding (%)	Sperm per ZP	n	ZP binding (%)	Sperm per ZP
A	Fresh electro-ejaculated	98	68^a1^	9.3 ± 20.6^a1^	113	28^b1^	1 ± 2.8^b1^
B	FT epididymal	51	27^a2^	2.7 ± 9.8^a2^	45	0^b2^	0 ± 0^b2^

^a-b ^Means with different superscripts indicate significant differences in ZP binding and number of spermatozoa per ZP between *in vitro *matured and FT oocytes (P < 0.05);^1–2 ^means with different superscripts indicate significant differences (P < 0.05) between fresh and FT spermatozoa.

**Figure 2:**
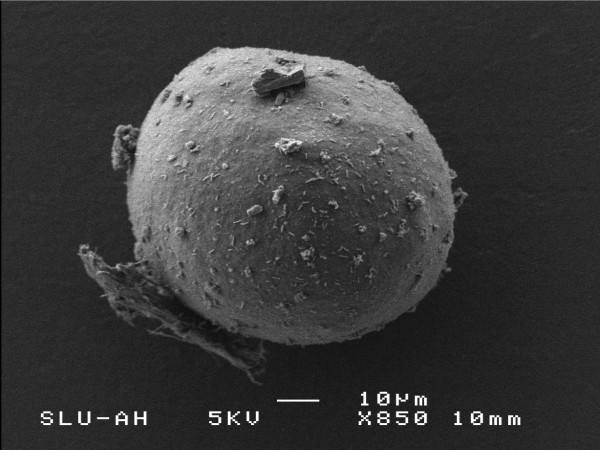
Scanning electron micrograph of the ZP of *in vitro *matured feline oocytes.

**Figure 3:**
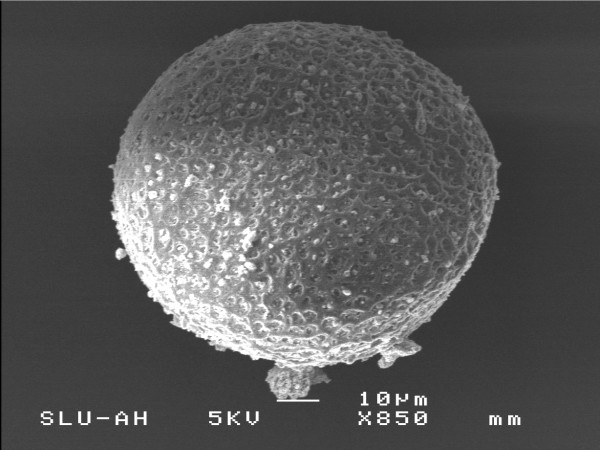
Scanning electron micrograph of the ZP of frozen-thawed feline oocytes. The size of the scale bar is 10 μm as shown in figures.

The ZP surface differed in morphology (Figure 2, 3). The *in vitro *matured oocytes showed a dense outer surface with few fenestrations (Figure 2), in contrast to the FT, immature oocytes, where fenestrations were conspicuously larger (Figure 3).

## Discussion

The results reveal that under the conditions of the present study, FT, immature ZP (oocytes) are less suitable for use in feline ZBA. Only a very low number of fresh spermatozoa bound to FT ZP, compared with their fresh, *in vitro *matured counterparts. With FT spermatozoa tested on FT ZP, there was no sperm-zona binding at all. This may partly be due to changes in the capacity of spermatozoa to maintain a normal plasma membrane surface that could bind to the specific receptors present in the ZP.

The spermatozoa used for the ZBA were cleansed prior to exposure to the oocytes. Two different procedures were used, washing and centrifugation or gradient centrifugation through a column of Percoll, processes that are not harmful to spermatozoa [15,30,33,34] but that are used to recover the best spermatozoa, in terms of motility and sperm viability [35]. As expected, the motility of electroejaculated fresh spermatozoa did not differ before and after the procedures, whereas a large variation was seen among the FT spermatozoa. It is not likely that the cleansing procedures, in particular using Percoll gradients, would have influenced the results of the ZBA, considering the very few FT spermatozoa that bound to *in vitro *matured ZP in comparison with freshly ejaculated spermatozoa.

Both epididymal, electroejaculated spermatozoa and spermatozoa collected by artificial vagina have been used for ZBAs in the domestic cat. A comparison of ZP attachment between epididymal and ejaculated spermatozoa in the domestic cat has revealed that more epididymal compared with ejaculated spermatozoa bind in the first 60 minutes following incubation, without differences in sperm capacitation status between them [10,36]. By contrast, a study on IVF of hamster ova found no differences regarding penetration rates, time of sperm penetration, and sperm concentration between ejaculated and epididymal spermatozoa [37]. To the authors' knowledge, corresponding work has not previously been performed in the domestic cat, and therefore we do not know how much the sperm source would have influenced the results of the ZBA in the present study. In any case, if epididymal spermatozoa should bind better than ejaculated spermatozoa, it should have influenced the results of FT spermatozoa in a positive way. However, there was no zona binding at all between FT oocytes and FT spermatozoa.

The incubation time for the sperm-oocyte complexes in the present study was based on Goodrowe & Hay's results [10]. These authors revealed that the number of attached sperm/zona and the percentage of zonae with attached spermatozoa reached maximum values after 4 hours of incubation, to decrease thereafter. Under the conditions of the present study, chilled spermatozoa bound to FT oocytes (data not shown) at a rate similar to that of fresh spermatozoa. These results correspond to those of Goodrowe & Hay [10], who found that chilled spermatozoa could be used for zona-free hamster ova penetration and homologous zona attachment at comparable rates as fresh spermatozoa. Our results clearly show a decreased, but not abolished, capacity of FT spermatozoa to bind to *in vitro *matured ZP. The viability of the FT spermatozoa used, assessed as progressive motility, was acceptable – albeit lower than that of fresh spermatozoa, even considering the use of cleansing procedures that would have selected for better sperm morphology, viability and motility. Such differences in viability between fresh and FT spermatozoa may explain the differences in ZP binding registered. However, the lack of binding to FT ZP is mainly due to the ZP and not to the spermatozoa, since fresh spermatozoa also had markedly decreased binding to FT ZP. In line with this argumentation, such decreased binding capacity of the ZP may reside in changes of the structure of the ZP that occur during unprotected freezing and/or thawing of the ovaries. The functional ability of the ZP to bind spermatozoa is closely related to its morphological appearance [38]. Oocyte storage can cause structural changes in the ZP, which may affect the number of bound spermatozoa [7].

However, in the female dog, the freezing of ovaries and zona binding with FT oocytes are possible, as previously indicated in several studies [6-8]. In the present study using SEM on queen oocytes, a clear morphological difference was shown in the ZP outer surface between *in vitro *matured and FT ZP. The *in vitro *matured ZP showed a dense surface with few fenestrations in contrast to their FT, immature counterparts, where fenestrations were conspicuously larger. These results agree with those previously reported by Ström Holst et al. [7] in the female dog. In vitro maturation has been associated with a more porous appearance in other species (e.g. mouse [39]). The ultrastructural changes in the FT oocytes were probably caused by damage during the freezing-thawing process, causing significantly reduced sperm binding capacity. Moreover, the ultrastructural difference accounted for the observation that few (fresh), or no (FT), spermatozoa bound to FT oocytes in the present study. Storage may affect oocytes from queen cats and female dogs in different ways, and results for female dogs may therefore not be accurate for cats. For instance, it was revealed by Ström Holst et al. [7] that in dogs, fresh oocytes bind significantly more spermatozoa than salt-stored oocytes do. These results differ from those reported by Andrews et al. [9], who found no difference in zona binding capacity between fresh, matured oocytes and oocytes that had been salt-stored for 1.5–24 weeks after maturation in the queen. It was shown by Ström Holst et al. [7] that in dogs, deep-freezing of the ovaries is better than salt storage of the oocytes. Corresponding results have not been shown for the cat. Studies by other authors have indicated that FT queen oocytes could be used for ZBA in cats. For instance, Kashiwazaki et al. [27], using immature FT oocytes, report binding of epididymal FT spermatozoa to frozen-thawed oocytes, a binding that we were unable to show. Unfortunately, in their study the number of bound spermatozoa per oocyte was not given, and there was no control group with fresh oocytes. The oocytes were, moreover, frozen in a different manner than in the present study, which may explain the differences in results. In our study the ovaries of ovario-hysterectomised queens were simply frozen in NaCl at ~-20°C, and the oocytes retrieved after thawing. By contrast, in the study by Kashiwazaki et al. [27] the oocytes were recovered before freezing and were frozen in 0.25 mL plastic straws with 1.5 M glycerol, conditions that make the collection and use of these oocytes less practical. Similarly, in the study by Luvoni & Pellizarri [26] the oocytes were recovered before freezing, and frozen in 0.5 mL straws with cryo-protectant. The freezing procedures described by Luvoni & Pellizarri [26] and Kashiwazaki et al. [27] may maintain the structure of the ZP, thus being beneficial for ulterior ZBA. In the present study the ovaries were frozen and the oocytes were not retrieved until after thawing of the ovaries, in order to make collection of material as practical as possible.

## Conclusion

The hypothesis that cat spermatozoa, either fresh or FT, could bind to homologous ZP of oocytes retrieved from FT queen ovaries to a similar extent as to the ZP of fresh, *in vitro *matured oocytes, proved false. Whatever the cause, under the conditions of the present study, ZP from immature oocytes from FT ovaries are less suitable for use in feline ZBA, in contrast to the situation in other species. Further studies are needed to explore the causes of our results.

## Competing interests

The author(s) declare that they have no competing interests.

## Authors' contributions

All authors participated in the design of the study. UH and EA collected the samples, and UH performed the ZBAs. UH drafted the manuscript. EA and BSH participated in the coordination of the study and helped to draft the manuscript. All authors read and approved the final manuscript.
